# Short-term changes in bodily pain and associated baseline factors in patients with fibromyalgia receiving paraprobiotic supplementation: a retrospective observational study

**DOI:** 10.3389/fmed.2026.1803753

**Published:** 2026-04-24

**Authors:** David Castro Corredor, Luis Ángel Calvo Pascual, María B. García-Moreno García

**Affiliations:** 1 Department of Rheumatology, Hospital General Universitario de Ciudad Real, Ciudad Real, Spain; 2 Instituto de Investigación Sanitaria de Castilla-La Mancha (IDISCAM), Toledo, Spain; 3 Department of Quantitative Methods, ICADE, Universidad Pontificia de Comillas, Madrid, Spain; 4 Institute for Research in Technology (IIT), Comillas Pontifical University, Madrid, Spain; 5 Department of Statistics, Econometrics, Operations Research, Business Organization, and Applied Economics, University of Córdoba, Córdoba, Spain

**Keywords:** artificial intelligence, dyslipidemia, fibromyalgia, generalized estimating equations, paraprobiotic supplementation

## Abstract

**Background:**

Fibromyalgia is characterized by heterogeneous pain trajectories, and short-term clinical improvement may vary across patients. We explored whether routinely collected baseline clinical variables were associated with short-term bodily pain improvement in a real-world cohort of patients with fibromyalgia receiving paraprobiotic supplementation.

**Methods:**

In this retrospective observational study, 86 women with fibromyalgia receiving paraprobiotic supplementation were followed for 2 months. Bodily Pain (BP) was assessed using the SF-36 at baseline, 1 month, and 2 months. The primary analysis used generalized estimating equations (GEE) with Gaussian family, identity link, and exchangeable working correlation structure, adjusted for age, years since diagnosis, body mass index (BMI), smoking status, hypertension, and dyslipidemia. Sensitivity analyses included alternative GEE working correlation structures, a linear mixed-effects model with patient-specific random intercept, and an ANCOVA model for BP at T2 adjusted for baseline BP.

**Results:**

In the primary analysis, time was significantly associated with SF-36 bodily pain (global Wald *p* = 0.0018). Compared with baseline, BP scores increased non-significantly at T1 (*β* = 1.92, 95% CI − 0.39 to 4.23; *p* = 0.103) and significantly at T2 (*β* = 6.95, 95% CI 3.12 to 10.78; *p* < 0.001). The contrast between T2 and T1 was also significant (*β* = 5.03, 95% CI 1.45 to 8.60; *p* = 0.006). Dyslipidemia was independently associated with lower BP scores (*β* = −4.95, 95% CI − 9.71 to −0.19; *p* = 0.041), whereas smoking showed a weaker borderline association. Findings were consistent across sensitivity analyses.

**Conclusion:**

In this exploratory real-world cohort, SF-36 bodily pain scores improved over a two-month follow-up, with the clearest change observed at two months. Dyslipidemia was the factor most consistently associated with lower bodily pain scores across analytic approaches. These findings should be interpreted cautiously given the observational design and absence of a control group, but they support further prospective controlled studies to clarify symptom trajectories and the role of metabolic factors in fibromyalgia.

## Introduction

Fibromyalgia is a chronic pain syndrome characterized by widespread musculoskeletal pain, fatigue, sleep disturbances, and cognitive and psychosocial symptoms, leading to substantial impairment in health-related quality of life. It predominantly affects women and is associated with marked clinical heterogeneity in symptom severity, disease course, and response to treatment (1). Despite advances in understanding its pathophysiology, fibromyalgia remains challenging to manage, and current therapeutic strategies are largely aimed at symptom control rather than disease modification.

Pain represents a central and disabling feature of fibromyalgia, with considerable inter-individual variability in both baseline severity and longitudinal trajectories. Longitudinal studies have shown that symptom burden at baseline is closely related to subsequent outcomes, while many patients experience persistent symptoms over time with only modest improvement (2, 3). Identifying baseline characteristics associated with short-term changes in pain may therefore help to better understand heterogeneity in symptom evolution and inform stratified approaches to management.

**Figure 1:**
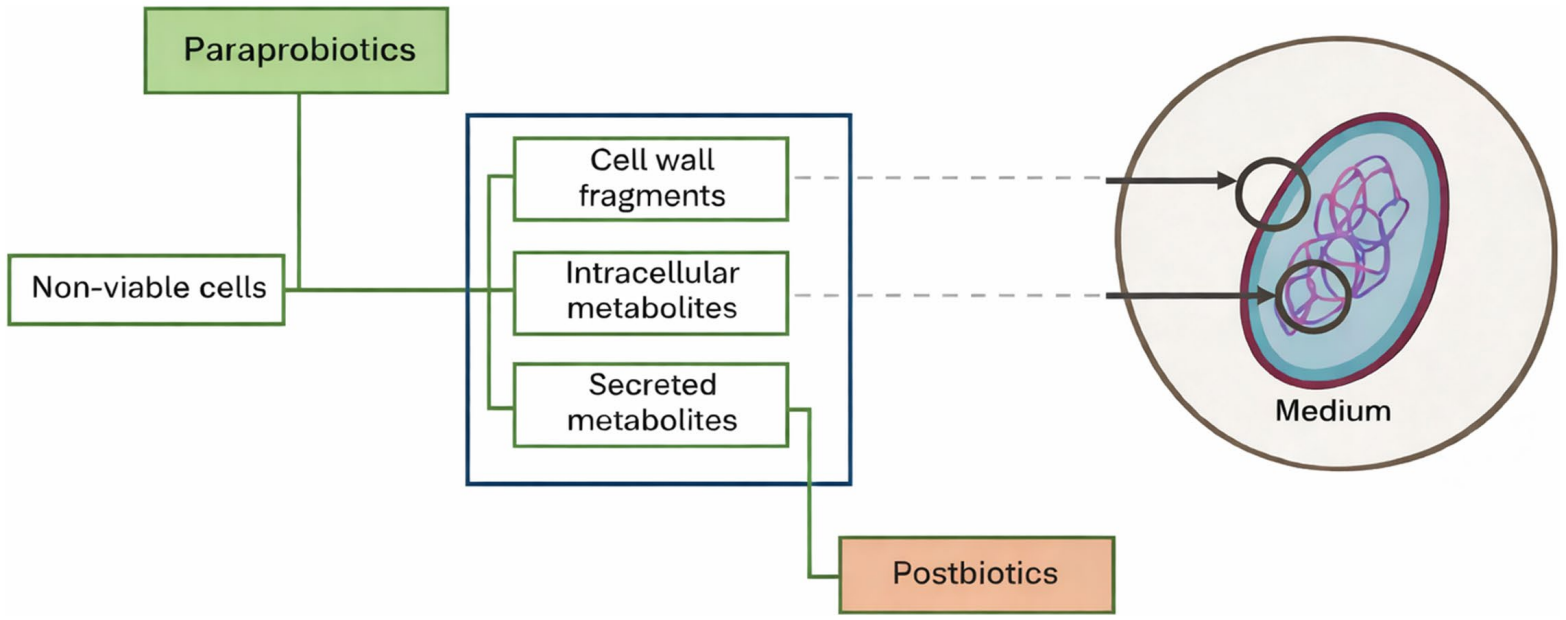
Schematic representation of a paraprobiotic and its derived components obtained from non-viable microbial cells.

Paraprobiotics are defined as non-viable microbial cells, cell fragments, or metabolic products derived from probiotic microorganisms that may confer health benefits to the host when administered in adequate amounts (4, 5). Unlike live probiotics, paraprobiotics do not require viability or colonization to exert biological effects, as their activity is mediated through structural components and bioactive metabolites (Figure 1). This concept is consistent with the International Scientific Association of Probiotics and Prebiotics (ISAPP) consensus on postbiotics, which recognizes non-viable microbial preparations as biologically active agents capable of modulating host responses (5). In this context, evaluating symptom trajectories in patients with fibromyalgia receiving paraprobiotic supplementation may help clarify whether these preparations merit further study in routine clinical practice.

Against this background, the short-term evolution of bodily pain in fibromyalgia during paraprobiotic supplementation remains insufficiently understood, particularly in routine clinical practice. Moreover, the baseline clinical and metabolic factors associated with pain trajectories in this context have not been well characterized. Accordingly, the aim of this retrospective observational study was to assess longitudinal changes in the SF-36 bodily pain domain over a two-month follow-up in patients with fibromyalgia receiving paraprobiotic supplementation, and to explore the association of baseline clinical and metabolic variables with bodily pain outcomes. The robustness of the findings was further examined through prespecified sensitivity analyses based on alternative longitudinal and baseline-adjusted models.

## Methods

### Participants

This was a retrospective observational study based on routinely collected clinical data. The data were obtained from electronic medical records of patients with fibromyalgia followed at the Rheumatology Department of the Hospital General Universitario de Ciudad Real (Spain). All variables were extracted from standardized clinical assessments performed as part of usual care and an observational research protocol. No data were collected specifically for the purposes of this analysis, and all information was anonymized before analysis in accordance with data protection regulations.

The study included 86 patients diagnosed with fibromyalgia who received paraprobiotic supplementation as part of an observational research protocol. Participants were recruited from clinical settings. All participants were informed about the objectives of the study and provided written informed consent prior to inclusion. Health-related quality of life was assessed using the SF-36 questionnaire at three time points over a two-month follow-up period: baseline (T0), 1 month (T1), and 2 months (T2). Only participants with complete longitudinal data on bodily pain outcomes were included in the analytical sample. Descriptive statistics (means and standard deviations) for all SF-36 items across the three assessment periods are provided in Supplementary File 1. Given the observational nature of the study, all analyses were exploratory and aimed at identifying predictive associations rather than causal effects.

### Measures

In addition to the SF-36 questionnaire, several clinical and treatment-related measures were collected. Continuous variables included age, body mass index, years since diagnosis of fibromyalgia, and age at diagnosis. Categorical variables comprised smoking status (non-smoker, former smoker, and current smoker), comorbidities (hypertension, dyslipidemia, diabetes mellitus, ischemic heart disease, digestive malabsorption, gluten intolerance, and lactose intolerance), daily alcohol consumption, current analgesic treatments (paracetamol/metamizole, non-steroidal anti-inflammatory drugs, tramadol, tapentadol, oxycodone, corticosteroids, and major opioids such as fentanyl or buprenorphine), psychotropic medications (selective serotonin reuptake inhibitors, serotonin–norepinephrine reuptake inhibitors, atypical antidepressants, benzodiazepines, pregabalin, and gabapentin), and engagement in psychological therapies. Medication-related variables captured use during the 6 months before baseline assessment. No missing data were observed for the variables included in the analysis. A detailed descriptive summary of all continuous and categorical variables is presented in Table 1.

**Table 1:** Baseline characteristics of the study population.

Categorical variables			
Group	Variable	Category	*n* (%)
Comorbidities	Hypertension	No	56 (65.1)
	Hypertension	Yes	30 (34.9)
	Dyslipidemia	No	51 (59.3)
	Dyslipidemia	Yes	35 (40.7)
	Diabetes mellitus	No	79 (91.9)
	Diabetes mellitus	Yes	7 (8.1)
	Ischemic heart disease	No	84 (97.7)
	Ischemic heart disease	Yes	2 (2.3)
	Digestive malabsorption	No	37 (43.0)
	Digestive malabsorption	Yes	49 (57.0)
	Gluten intolerance	No	78 (90.7)
	Gluten intolerance	Yes	8 (9.3)
	Lactose intolerance	No	51 (59.3)
	Lactose intolerance	Yes	33 (38.4)
Lifestyle factors	Daily alcohol consumption	No	82 (95.3)
	Daily alcohol consumption	Yes	4 (4.7)
Analgesics	Paracetamol/Metamizole	No	28 (32.6)
	Paracetamol/Metamizole	Yes	58 (67.4)
	NSAIDs	No	40 (46.5)
	NSAIDs	Yes	46 (53.5)
	Tramadol	No	61 (70.9)
	Tramadol	Yes	24 (27.9)
	Tapentadol	No	73 (84.9)
	Tapentadol	Yes	13 (15.1)
	Oxycodone	No	80 (93.0)
	Oxycodone	Yes	6 (7.0)
	Corticosteroids	No	61 (70.9)
	Corticosteroids	Yes	24 (27.9)
	Major opioids (fentanyl/buprenorphine)	No	81 (94.2)
	Major opioids (fentanyl/buprenorphine)	Yes	5 (5.8)
Psychotropic medication	Antidepressants (SSRIs)	No	65 (75.6)
	Antidepressants (SSRIs)	Yes	21 (24.4)
	Antidepressants (SNRIs)	No	56 (65.1)
	Antidepressants (SNRIs)	Yes	30 (34.9)
	Antidepressants (atypical)	No	62 (72.1)
	Antidepressants (atypical)	Yes	24 (27.9)
	Benzodiazepines	No	69 (80.2)
	Benzodiazepines	Yes	17 (19.8)
	Neuromodulators (pregabalin)	No	67 (77.9)
	Neuromodulators (pregabalin)	Yes	18 (20.9)
	Neuromodulators (gabapentin)	No	77 (89.5)
	Neuromodulators (gabapentin)	Yes	8 (9.3)
Psychological therapies	Psychological therapies	No	53 (61.6)
	Psychological therapies	Yes	33 (38.4)

Continuous variables		
Variables	Mean (SD)	Median [IQR]
Age (years)	54.9 (10.2)	56.0 [49.2–61.0]
Body mass index (kg/m^2^)	26.0 (5.1)	25.8 [22.1–28.7]
Years since diagnosis (years)	13 (11)	10 [4–20]
Age at diagnosis (years)	42.3 (10.8)	43.5 [34.0–50.0]

Categorical variables are reported as *n* (%), and continuous variables as mean (SD) and median [IQR].

**Figure 2:**
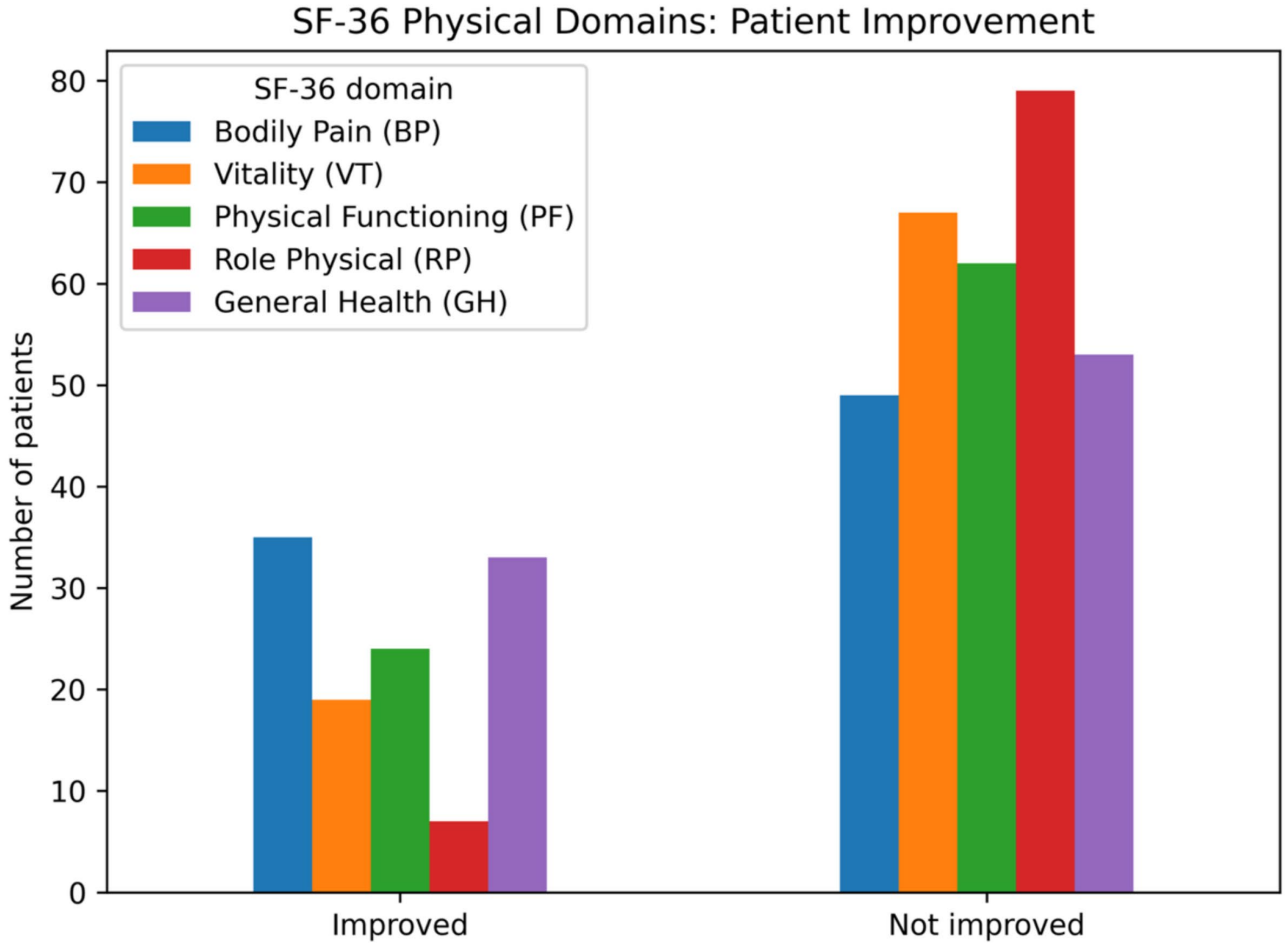
Number of patients classified as improved or not improved across SF-36 physical health domains based on longitudinal trajectories from baseline (T0), intermediate follow-up (T1), and final follow-up (T2).

The main outcome for the primary analyses was the Bodily Pain (BP) domain of the SF-36 questionnaire, assessed longitudinally at baseline (T0), 1 month (T1), and 2 months (T2). BP was derived from items 21 and 22 of the SF-36 by linearly transforming the corresponding item responses to a 0–100 scale and averaging them, with higher scores indicating less pain. Accordingly, the primary analyses examined changes in BP scores over time rather than a binary improvement outcome. For descriptive purposes only, patients were additionally classified as improved or not improved across selected SF-36 physical health domains for graphical display in Figure 2.

### Quantitative analysis

BP scores were calculated by linearly transforming the corresponding 21 and 22 SF-36 items to a 0–100 scale. Longitudinal changes were analyzed using generalized estimating equations (GEE) with Gaussian family, identity link, and an exchangeable working correlation structure to account for within-subject repeated measures. Time was modeled as a categorical variable (reference: T0), and the analysis was adjusted for baseline age, years since diagnosis, body mass index, smoking status, hypertension, and dyslipidemia. Continuous covariates were mean-centered. Complete-case analysis was performed, and robust standard errors were used for inference. Overall effects were assessed with Wald tests, and a linear contrast was used to compare T2 versus T1.

As a first sensitivity analysis, the robustness of the primary GEE results to the choice of working correlation structure was assessed by refitting the same model under three alternative specifications: independence, exchangeable, and first-order autoregressive (AR-1). The same analytic dataset, outcome definition, and covariate adjustment used in the primary analysis were retained. Model fit was compared using the quasi-likelihood under the independence model criterion (QIC), and the consistency of parameter estimates, standard errors, and statistical significance across correlation structures was evaluated.

As a second sensitivity analysis, the primary longitudinal model was re-estimated using a linear mixed-effects model with a patient-specific random intercept. The model included a random intercept for the patient to account for correlation among repeated observations within the same individual. Before fitting, the fixed-effects design matrix was evaluated to confirm full rank and exclude rank-deficiency problems. To improve numerical stability and ensure convergence, several optimization strategies were prespecified and sequentially attempted when needed, including different optimization algorithms (limited-memory BFGS, BFGS, conjugate gradient, Powell, and Nelder–Mead) and both restricted maximum likelihood (REML) and maximum likelihood (ML) estimation.

As a third sensitivity analysis, an analysis of covariance (ANCOVA) model was fitted to evaluate the association between baseline characteristics and the SF-36 bodily pain score at T2 while adjusting for the corresponding baseline score at T0. The outcome variable was BP_T2, and BP_T0 was included as a covariate to account for baseline differences in bodily pain. The model retained the same baseline covariates used in the primary analysis, namely age, years since diagnosis, body mass index, smoking status, hypertension, and dyslipidemia. Continuous covariates were mean-centered, smoking status was modeled as a three-category variable, and hypertension and dyslipidemia were entered as binary variables. The same complete-case dataset and the same 0–100 scoring procedure for the SF-36 bodily pain domain were used. The model was estimated using ordinary least squares regression with HC3 heteroscedasticity-robust standard errors.

All statistical analyses were performed in Python, using pandas and NumPy for data processing, statsmodels for GEE, linear mixed-effects, and ANCOVA modeling, patsy for design matrix diagnostics, and Matplotlib for graphical visualization.

## Results

Baseline descriptive characteristics of the study population are summarized in Table 1. The median body mass index was 25.8 kg/m^2^ (IQR 22.1–28.7), and the median time since fibromyalgia diagnosis was 10 years (IQR 4–20). Diabetes mellitus was present in 8.1% of participants, while 27.9% reported corticosteroid use during the 6 months prior to baseline assessment. Figure 2 depicts the longitudinal evolution of SF-36 dimensions across the study period. With respect to bodily pain, 36 participants showed improvement, whereas 50 did not improve over follow-up.

**Figure 3:**
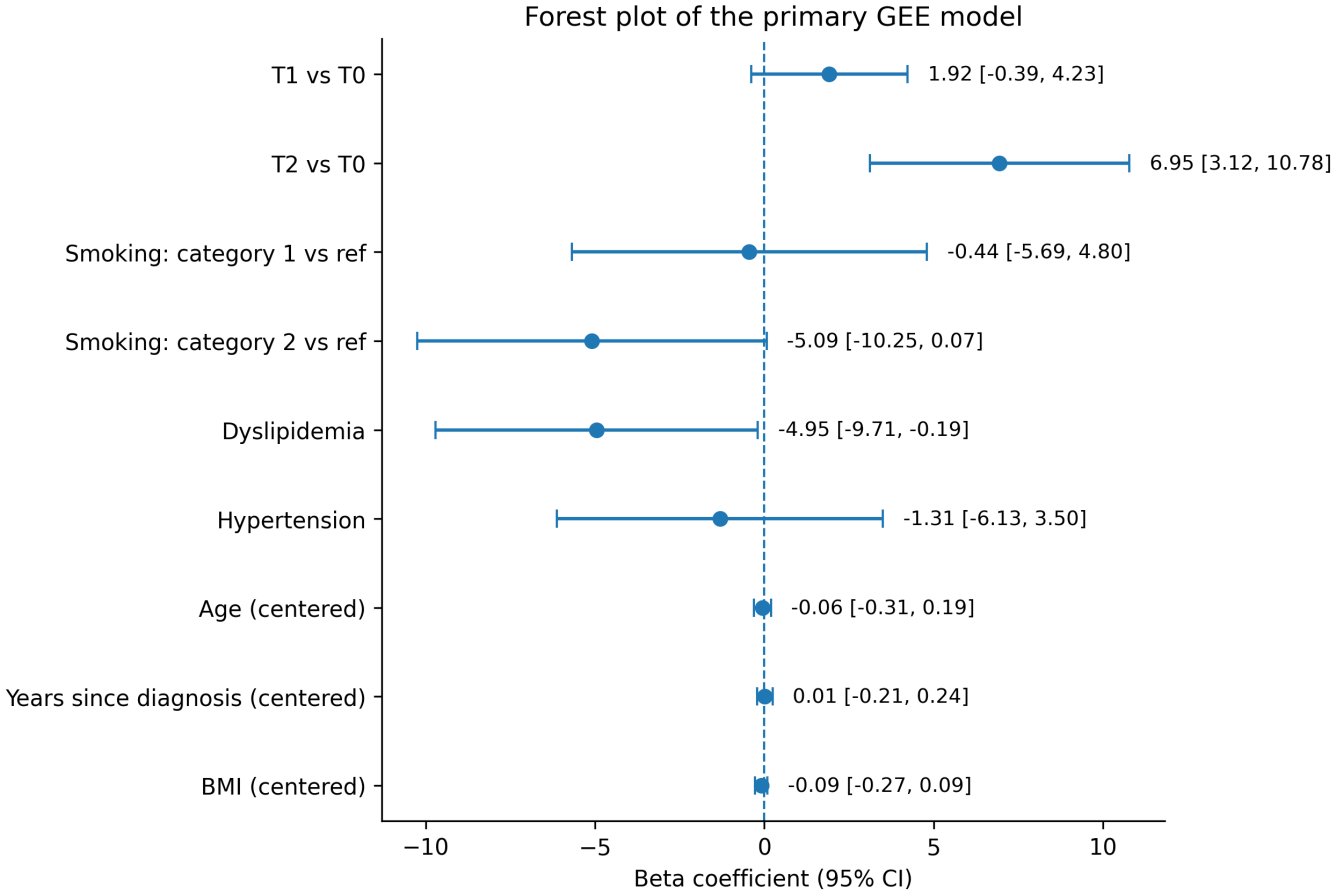
Adjusted associations between time, clinical covariates, and SF-36 bodily pain: results from the primary GEE model.

**Figure 4:**
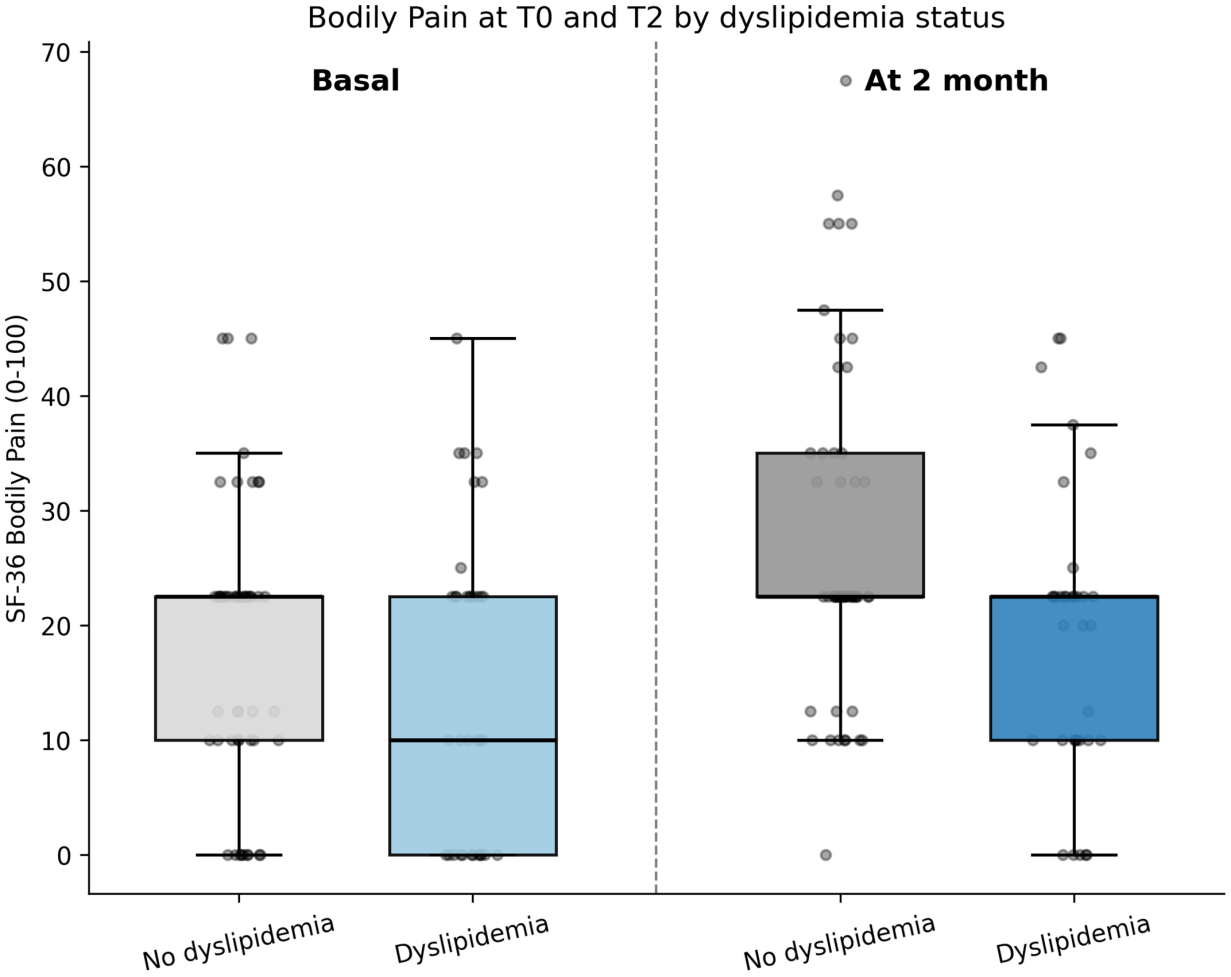
Distribution of SF-36 bodily pain scores at baseline and 2-month follow-up according to dyslipidemia status.

The primary analysis, based on the adjusted GEE model, showed a significant overall association between time and SF-36 bodily pain (global Wald *p* = 0.0018). Compared with baseline, BP scores increased non-significantly at T1 (*β* = 1.92, 95% CI − 0.39 to 4.23; *p* = 0.103) and significantly at T2 (*β* = 6.95, 95% CI 3.12 to 10.78; *p* < 0.001). The contrast between T2 and T1 also showed a significant additional improvement at T2 (*β* = 5.03, 95% CI 1.45 to 8.60; *p* = 0.006). Among covariates, dyslipidemia was independently associated with lower BP scores (*β* = −4.95, 95% CI − 9.71 to −0.19; *p* = 0.041), while smoking category 2 showed a borderline association with worse BP (*β* = −5.09; *p* = 0.053). No significant associations were found for hypertension, age, years since diagnosis, or body mass index. Additional information is provided in Supplementary File 2. A graphical summary of the adjusted coefficients from the primary model is shown in Figure 3 (forest plot), and the distribution of bodily pain scores at T0 and T2 according to dyslipidemia status is presented in Figure 4.

In the first sensitivity analysis, results were virtually identical across all working correlation structures, with no meaningful differences in parameter estimates, standard errors, or statistical significance. The estimated effects of time remained unchanged, with a non-significant increase at T1 and a significant improvement at T2 (*β* = 6.95, *p* < 0.001), as well as a consistent difference between T2 and T1 (*β* = 5.03, *p* = 0.006) in all models. Global tests for time and covariates were also unchanged across specifications. Model fit, as assessed by QIC, showed only minimal differences between structures, with a slightly lower value for the AR-1 model (QIC = 274.25) than for the independence and exchangeable structures (QIC = 275.96). Additional details of this first sensitivity analysis are provided in Supplementary Table 1.

In the second sensitivity analysis, the linear mixed-effects model included 258 observations from 86 patients and converged successfully. Compared with baseline, BP scores increased by 1.92 points at T1 (95% CI − 1.41 to 5.24; *p* = 0.258) and by 6.95 points at T2 (95% CI 3.62 to 10.27; *p* < 0.001). For smoking status, category 1 showed a coefficient of −0.44 (95% CI − 8.04 to 7.15; *p* = 0.909), and category 2 showed a coefficient of −5.09 (95% CI − 10.85 to 0.67; *p* = 0.083), both relative to the reference category. Dyslipidemia had a coefficient of −4.95 (95% CI − 9.63 to −0.28; *p* = 0.038), whereas hypertension had a coefficient of −1.31 (95% CI − 6.42 to 3.80; *p* = 0.614). The coefficients for age, years since diagnosis, and body mass index were −0.06 (95% CI − 0.32 to 0.20; *p* = 0.660), 0.01 (95% CI − 0.22 to 0.24; *p* = 0.912), and −0.09 (95% CI − 0.26 to 0.08; *p* = 0.287), respectively. The estimated random-intercept variance was 71.15 and the residual variance was 123.81. Additional details of this second sensitivity analysis are provided in Supplementary Table 2.

In the third sensitivity analysis, the ANCOVA model included 86 patients. The coefficient for baseline bodily pain (BP_T0) was 0.03 (95% CI − 0.22 to 0.28; *p* = 0.810). For smoking status, category 1 showed a coefficient of 3.17 (95% CI − 10.42 to 16.75; *p* = 0.648), and category 2 showed a coefficient of −4.37 (95% CI − 11.92 to 3.17; *p* = 0.256), both relative to the reference category. Dyslipidemia had a coefficient of −7.70 (95% CI − 14.12 to −1.27; *p* = 0.019), whereas hypertension had a coefficient of 0.93 (95% CI − 6.45 to 8.30; *p* = 0.806). The coefficients for age, years since diagnosis, and body mass index were −0.14 (95% CI − 0.52 to 0.24; *p* = 0.477), −0.08 (95% CI − 0.40 to 0.25; *p* = 0.648), and −0.15 (95% CI − 0.44 to 0.14; *p* = 0.308), respectively. The model *R*^2^ was 0.124 and the adjusted *R*^2^ was 0.033. Additional details of this third sensitivity analysis are provided in Supplementary Table 3.

## Discussion

The present retrospective observational study yielded two main findings. First, SF-36 bodily pain scores improved over follow-up, with the clearest separation observed at 2 months rather than at 1 month. Second, dyslipidemia was the factor most consistently associated with lower bodily pain scores, whereas smoking showed only a weaker borderline association and BMI, age, years since diagnosis, and hypertension were not independently associated. These findings are clinically relevant because bodily pain is among the SF-36 domains most severely affected in fibromyalgia and contributes substantially to impaired quality of life. In a Spanish cohort, Fernandez-Feijoo et al. (6) reported particularly low SF-36 scores in the bodily pain and vitality domains. At the same time, longitudinal studies suggest that fibromyalgia trajectories are heterogeneous and that spontaneous improvement is generally modest. Walitt et al. (2) described persistent symptom burden with only slight average improvement over time, and Schaefer et al. (7) similarly reported symptom fluctuation rather than uniform recovery. Against this background, the delayed improvement observed in our cohort is of interest, although it should be interpreted cautiously.

Since all participants received paraprobiotic supplementation, the present data do not allow any inference regarding treatment efficacy. Nevertheless, the observed pattern is compatible with a growing literature implicating the gut-brain axis in fibromyalgia. Minerbi et al. (4) described alterations in gut microbiome composition in fibromyalgia, including changes associated with clinical manifestations. However, interventional evidence remains preliminary and inconsistent. In a pilot randomized trial, Roman et al. (8) reported improvements in some cognitive and emotional outcomes, but not clear superiority for core physical fibromyalgia manifestations, whereas Calandre et al. (9) did not demonstrate efficacy of VSL#3 for gastrointestinal or fibromyalgia-related symptomatology. Taken together, these findings support further investigation of microbiota-targeted strategies, while not supporting a causal interpretation that paraprobiotic supplementation itself accounted for the observed improvement.

One of the most consistent associations observed in our analyses was that between dyslipidemia and lower bodily pain scores. This finding is in line with a cardiometabolic perspective on fibromyalgia heterogeneity. Cordero et al. (10) reported that clinical symptoms in fibromyalgia were associated with overweight and lipid profile, while Rus et al. (11) found abnormalities in nitric oxide, inflammatory markers, lipid profile, and cortisol in women with fibromyalgia, particularly when stratified by body weight. At a broader syndrome level, Yahia et al. (12) reported a high prevalence of metabolic syndrome in newly diagnosed fibromyalgia and more severe symptom profiles in patients with metabolic syndrome. Although the underlying mechanism cannot be established in the present study, dyslipidemia may be a marker of a more symptomatic subgroup or of inflammatory-metabolic processes relevant to pain persistence.

The absence of an independent association between BMI and bodily pain after adjustment is also informative. Several studies have linked higher BMI or less favorable body composition to worse fibromyalgia severity and poorer quality of life (13–15). However, these associations have not been entirely consistent across cohorts, and our results suggest that, in some settings, metabolic profile may be more informative than BMI alone. One possible explanation is that BMI and dyslipidemia capture related but non-equivalent dimensions of cardiometabolic burden. Another is that the modest sample size reduced power to detect a smaller adjusted BMI effect.

The association observed for smoking was weaker and should be interpreted with caution. In our study, the former-smoker category showed only a borderline association with lower bodily pain scores, whereas the strongest evidence in the literature relates to current smoking. Previous studies have associated smoking with worse fibromyalgia symptoms (16), greater pain and interference in chronic pain populations with fibromyalgia-like symptomatology (17), and higher widespread pain and symptom severity in patients with fibromyalgia (18). Accordingly, the borderline finding observed here may reflect cumulative exposure, residual confounding, or limited subgroup size rather than a robust former-smoker effect.

A major strength of this study is the consistency of the findings across multiple analytic approaches. The time effect and the negative association with dyslipidemia were materially similar in the primary GEE model, across alternative working-correlation structures, in the patient-specific mixed-effects model, and in the baseline-adjusted ANCOVA. This convergence reduces the likelihood that the findings were driven by a single modeling assumption and supports the robustness of the overall pattern. More broadly, these results are consistent with an increasing recognition that outcomes in rheumatic diseases are shaped by a multidimensional interplay of clinical, metabolic, and contextual factors (19–21).

Several limitations should be acknowledged. First, the retrospective observational design and the absence of a control group preclude causal inference; therefore, the improvement observed over time may reflect natural symptom fluctuation, nonspecific follow-up effects, concomitant interventions, or unmeasured clinical changes rather than a specific effect of paraprobiotic supplementation. Second, the sample size was modest and derived from a single center, which may limit precision and external validity. Third, follow-up was limited to 2 months, whereas previous longitudinal studies have shown that fibromyalgia trajectories remain variable over longer periods (2, 7). Fourth, although the models adjusted for several clinically relevant covariates, residual confounding cannot be excluded, particularly with respect to disease severity, medication changes, physical activity, diet, and psychosocial context. This is especially relevant in fibromyalgia, where symptom burden is not explained exclusively by biological variables. In this regard, Ortega-Martínez et al. (22) reported that socio-affective factors, including perceived loneliness and socio-family context, were associated with the impact of fibromyalgia on patients’ lives, reinforcing the view that broader contextual determinants may contribute meaningfully to pain burden.

Overall, the present study suggests that bodily pain improved over the two-month observation period and that dyslipidemia may help identify patients with a less favorable pain profile. In view of the mixed results of prior probiotic trials (8, 9), the observational microbiome evidence (4), and the cardiometabolic associations described in fibromyalgia (10–12), the next step should be a prospective controlled study with longer follow-up and predefined metabolic stratification.

## Conclusion

In conclusion, this retrospective observational study suggests that SF-36 bodily pain scores improved over a two-month follow-up period in patients with fibromyalgia receiving paraprobiotic supplementation, with the clearest change observed at 2 months. Dyslipidemia was the factor most consistently associated with lower bodily pain scores across the different analytic approaches. Although the observational design and absence of a control group preclude causal inference, the consistency of the findings across the primary analysis and sensitivity analyses supports the robustness of the overall pattern. These results highlight the potential relevance of metabolic factors in fibromyalgia-related pain and support the need for prospective controlled studies with longer follow-up to clarify the role of paraprobiotic strategies and to identify patient subgroups with different symptom trajectories.

## Data Availability

The original contributions presented in the study are included in the article/Supplementary material, further inquiries can be directed to the corresponding author.

## Supplementary material

Supplementary File 1 Descriptive statistics (means and standard deviations) for all SF-36 items across the three assessment periods.

Supplementary File 2 Additional information from the primary GEE model.

Supplementary Table 1 Additional details of the first sensitivity analysis (alternative GEE working correlation structures).

Supplementary Table 2 Additional details of the second sensitivity analysis (linear mixed-effects model).

Supplementary Table 3 Additional details of the third sensitivity analysis (ANCOVA model).
